# Evaluation of Swallow Function in Patients with Craniofacial Microsomia: A Retrospective Study

**DOI:** 10.1007/s00455-017-9851-x

**Published:** 2017-11-04

**Authors:** Lara S. van de Lande, Cornelia J. J. M. Caron, Britt. I. Pluijmers, Koen F. M. Joosten, Marloes Streppel, David J. Dunaway, Maarten J. Koudstaal, Bonnie L. Padwa

**Affiliations:** 1 The Dutch Craniofacial Center, Department of Oral and Maxillofacial Surgery, Erasmus MC, Sophia Children’s Hospital Rotterdam, ’s Gravendijkwal 230, 3015 CE Rotterdam, The Netherlands; 2 The Dutch Craniofacial Center, Department of Ear, Nose and Throat Surgery, Erasmus MC, Sophia Children’s Hospital, Rotterdam, The Netherlands; 3 The Craniofacial Unit, Great Ormond Street Hospital, London, UK; 4 Department of Plastic and Oral Surgery, Boston Children’s Hospital, Boston, USA

**Keywords:** Craniofacial microsomia, Hemifacial microsomia, Feeding difficulties, Dysphagia, Modified barium swallow test, Swallow function

## Abstract

Craniofacial microsomia (CFM) is characterized by underdevelopment of the structures derived from the first and second pharyngeal arches resulting in aesthetic, psychological, and functional problems including feeding and swallowing difficulties. The aim of this study is to gain more insight into swallowing difficulties in patients with CFM. A retrospective study was conducted in the population of patients diagnosed with CFM at three major craniofacial units. Patients with feeding difficulties and those who underwent video fluoroscopic swallow (VFS) studies were included for further analyses. The outcome of the VFS-studies was reviewed with regard to the four phases of swallowing. In our cohort, 13.5% of the 755 patients were diagnosed with swallowing difficulties. The outcome of the VFS-studies of 42 patients showed difficulties in the oral and pharyngeal phases with both thin and thick liquids. Patients with more severe mandibular hypoplasia showed more difficulties to form an appropriate bolus compared to patients who were less severely affected. This is the first study to document swallowing problems in patients with CFM. Difficulties were seen in both the oral and pharyngeal phases. We recommend routine screening for swallowing issues by a speech and language therapist in all patients with CFM and to obtain a VFS-study in patients with a type III mandible.

**Electronic supplementary material:**

The online version of this article (doi:10.1007/s00455-017-9851-x) contains supplementary material, which is available to authorized users.

## Introduction

Craniofacial microsomia (CFM) is a complex and heterogeneous condition characterized by underdevelopment of structures derived from the first and second pharyngeal arches including the orbit, mandible, ear, facial nerves, facial soft tissues, and muscles [1, 2]. The most striking feature, mandibular hypoplasia, is present in 89 to 100% of the patients. With an incidence of 1:3000 to 1:5000 live births, CFM is believed to be the second most common craniofacial anomaly following cleft lip and palate [2–4].

The facial anomalies seen in CFM may not only lead to aesthetic and psychological problems, but also to functional issues such as breathing and feeding difficulties (FD) [5, 6]. FD are seen in 42–83% of the patients with CFM and include problems with suckling, chewing, failure to thrive, and swallowing [5, 7–9].

Feeding and swallowing are complex neuromuscular functions that are dependent upon volitional and reflexive activities of a significant number of oropharyngeal muscles and nerves that form the oropharyngeal apparatus. Reflexive activities play a dominant role up to 6 months in healthy infants [10–13].

Normal swallowing is divided into four phases that proceed seamlessly from one to another for which adequate neuromuscular coordination is necessary. During the four phases of swallowing (i.e., preparatory, oral, pharyngeal, and esophageal), the bolus is formed and transported into the stomach via the oropharynx and esophagus [10, 14–17]. To evaluate the different phases of swallowing, a videofluoroscopic swallow study (VFS-study) can be used, which is considered to be the gold standard [18–20]. With this imaging technique, all four phases of swallowing can be assessed using pellets of different consistencies, e.g., thin liquids, thick liquids, purees, and solids.

Swallow difficulties (SD) can result from a wide variety of functional or structural deficits of the oral cavity, pharynx, larynx, or esophagus [10]. SD in CFM might be the result of mandibular hypoplasia, possible underdevelopment of the oropharyngeal apparatus, and/or decreased innervation of the masticatory and pharyngeal muscles [7, 11, 21]. Furthermore, swallow dysfunction might be aggravated by cleft lip and/or palate, which is present in 15.9% of the patients with CFM [22–24].

The aim of this study is to document the incidence of SD in patients with CFM and gain more insight into SD in patients with CFM by studying the outcomes of VFS-studies at three major craniofacial units.

## Materials and methods

A retrospective study was conducted in the population of patients diagnosed with CFM at the craniofacial units of Erasmus MC, Rotterdam, The Netherlands; Great Ormond Street Hospital in London, United Kingdom; and Boston Children’s Hospital in Boston, United States of America. Following IRB approval (Rotterdam: MEC-2013-575; London: 14DS25; Boston: X05-08-058), medical charts were reviewed for information on sex, affected side, severity of the deformity according to the Pruzansky–Kaban classification [4, 25], presence of FD and type of FD, presence of cleft lip and/or palate, cleft repair, presence of tracheostomy, reports of performed VFS-studies, and available clinical pictures and/or radiographic images (i.e., panoramic X-rays and/or CT head). Patients with and without cleft (lip) palate were independently analyzed.

Charts of patients with documented FD were reviewed for type of FD, i.e., swallow difficulties. FD were clinically determined by the treating physician. Patients clinically diagnosed with SD who had undergone a VFS-study were included for further analyses. The criteria used to determine SD are described in Table 1.

**Table 1:** Criteria to determine swallow difficulties

Criteria swallow difficulties
Sucking and swallowing incoordination
Weak suck
Excessive gagging
Recurrent coughing during feeds
Recurrent pneumonia
Nasopharyngeal reflux
Desaturation during feeds
(Risk for) aspiration during feeds

Original reports of all VFS-studies were collected. Incomplete reports of the VFS-studies and VFS-studies performed following mandibular reconstruction were excluded. The first VFS-study per patient was used for (statistical) analyses. Information was collected on the number of performed VFS-studies; indication; age at time of the first VFS-study; positioning, seating, and imaging view during the VFS-study; nutritional route at time of the VFS-study (i.e., fully oral, oral in combination with a nasogastric tube, or completely fed by a nasogastric tube); and utensils used (e.g., bottle, spoon, nipple). When patients were fully fed via a nasogastric tube at time of the VFS-study, the VFS-study was nevertheless fully orally assessed. Information on the outcome of the VFS-studies regarding the four phases of swallowing was collected. Impairment of the oral phase included impaired bolus formation and premature spill of the bolus into the pharynx. Premature spill of the bolus into the pharynx was defined as progression of the bolus over the tongue base into the pyriform sinus in the absence of purposeful oral transfer before the initiation of swallowing [26]. Bolus formation was tested with all four consistencies, whereas premature spill into the pharynx was only evaluated with thin and thick liquids. Impairment of the pharyngeal phase included delayed swallow trigger, post-swallow stasis, nasopharyngeal reflux, laryngeal penetration, and aspiration. Laryngeal penetration is defined as food/liquid passing the laryngeal inlet above the level of the vocal folds, whereas aspiration is defined as food/liquid passing the laryngeal inlet below the vocal folds, with or without the trigger for cough [26]. The esophageal phase included data on adequate movement of the bolus into the esophagus. Gastroesophageal reflux was not studied. The pharyngeal phase was evaluated using pellets with different consistencies, i.e., thin liquids, thick liquids, puree, and solids [10, 14–16, 27, 28].

Severity of mandibular hypoplasia in CFM was scored on panoramic X-rays or on CT scans according to the Pruzansky–Kaban classification. In patients with bilateral CFM, the Pruzansky–Kaban classification was scored on both sides of the patient; however, for analyses the most severe score was used.

### Statistical Analysis

Statistical analyses were performed using Statistical Package for Social Sciences (SPSS) version 20.0 for Windows (2011, SPSS Inc., Chicago, IL, USA). Descriptive statistics were used. Equality of groups was tested with the Pearson *χ* ^2^ test and Fisher’s Exact test. A *p* value of < 0.05 was considered statistically significant.

## Results

### Population

**Fig. 1:**
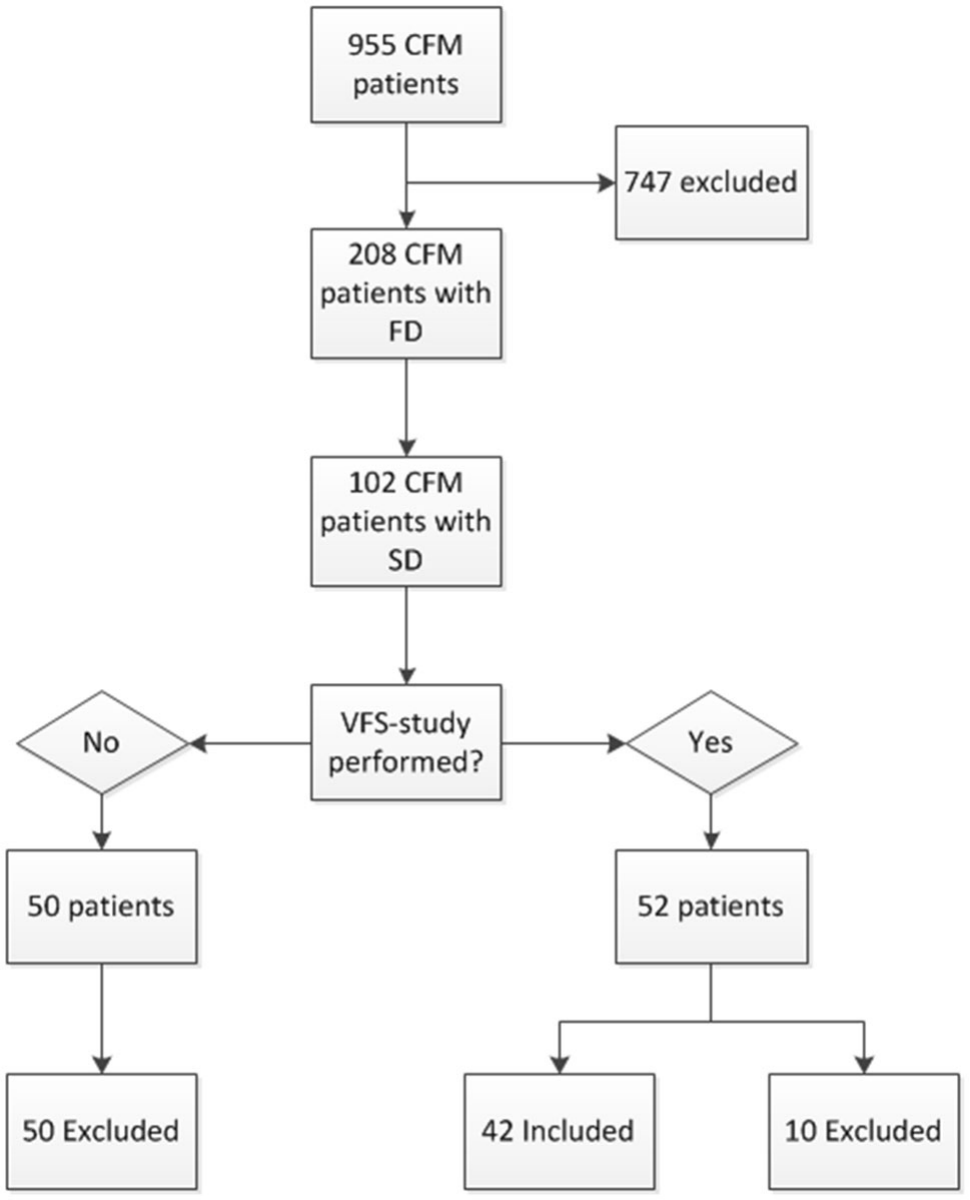
Inclusion and exclusion of patients with CFM and VFS-studies. *CFM* craniofacial microsomia, *SD* swallow difficulties, *FD* feeding difficulties, *VFS-study* videofluoroscopic swallow study

Of the 955 patients diagnosed with CFM, clinical pictures and/or radiographic images were available in 755 patients, who could be further reviewed and analyzed. In total, 208 patients were diagnosed with FD, of which 102 patients were diagnosed with SD. Of these patients, 51.0% had undergone a VFS-study. As there were no clinical concerns for aspiration, 50 patients did not undergo a VFS-study. Ten patients were excluded since the first available VFS-study was done following mandibular reconstruction. A total of 42 patients were included. Indications for the VFS-study were to assess function and safety of swallowing (*n* = 36), including the risk for (silent) aspiration (*n* = 4), or in case of excessive gagging and vomiting (*n* = 2) (Fig. 1).

### Characteristics of the VFS-Study Group

The study group consisted of 24 (57.1%) males and 18 (42.9%) females. In total, 31 (73.8%) patients were unilaterally and 11 (26.2%) patients were bilaterally affected. The Pruzansky–Kaban classification could be assessed in 31 patients, in which most patients were classified as Pruzansky–Kaban III (Table 2).

**Table 2:** Description of the included population

	No. of patients
Sex	
Male	24
Female	18
Laterality	
Unilateral CFM	31
Bilateral CFM	11
Affected side*	
Right side	19
Left side	12
P–K classification	
P–K I	9
P–K IIA	5
P–K IIB	6
P–K III	11
Unknown	11
Cleft lip/palate	
Cleft palate	8
Cleft lip and palate	4
Submucous cleft	1
No	29
Tracheostomy during VFS-study	
Cuffed	4
Uncuffed	2
History of tracheostomy	4
No tracheostomy	32

*CFM* craniofacial microsomia, *P–K classification* Pruzansky–Kaban classification

* In the unilateral cases of craniofacial microsomia

Cleft (lip) palate was diagnosed in 13 patients (31.0%); at time of the VFS-study, cleft (lip) palate was repaired in seven patients and unrepaired in three. In another three patients, the status of cleft (lip) palate repair remained unknown.

Six out of 42 patients had a tracheostomy at time of the VFS-study (Table 2).

All VFS-studies were performed in an upright position in a tumble forms feeder seat. Lateral view was standard. The oral and pharyngeal phases were tested in 41 and 42 patients, respectively. At time of the VFS-study, 25 patients were fully orally fed, six patients were nasogastric tube dependent, and 11 patients were fed both orally and via a nasogastric tube. Patients with cleft (lip) palate were significantly more often fed using a nasogastric tube at time of the VFS-study than patients without cleft (lip) palate (Pearson’s *χ* ^2^ (2) = 6.499, *p* = 0.039) (Table 3).

**Table 3:** Current nutritional route in patients with and without cleft lip/palate at time of the VFS-study

	Current nutritional route			Total
	Oral	Oral and NG tube	NG tube	
Cleft (lip) palate				
No	21	5	3	29
Yes	4	4	4	13
Total	25	11	6	42

*NG tube* nasogastric tube

Overall, the median age at time of the VFS-study was 1.15 years (range 0.02–26.26). A VFS-study was performed in 26.2% of patients before the age of 6 months. There were no (significant) differences between patients younger and older than 6 months regarding clinical features, such as severity of CFM, presenting symptoms, and indication for a VFS-study.

**Fig. 2:**
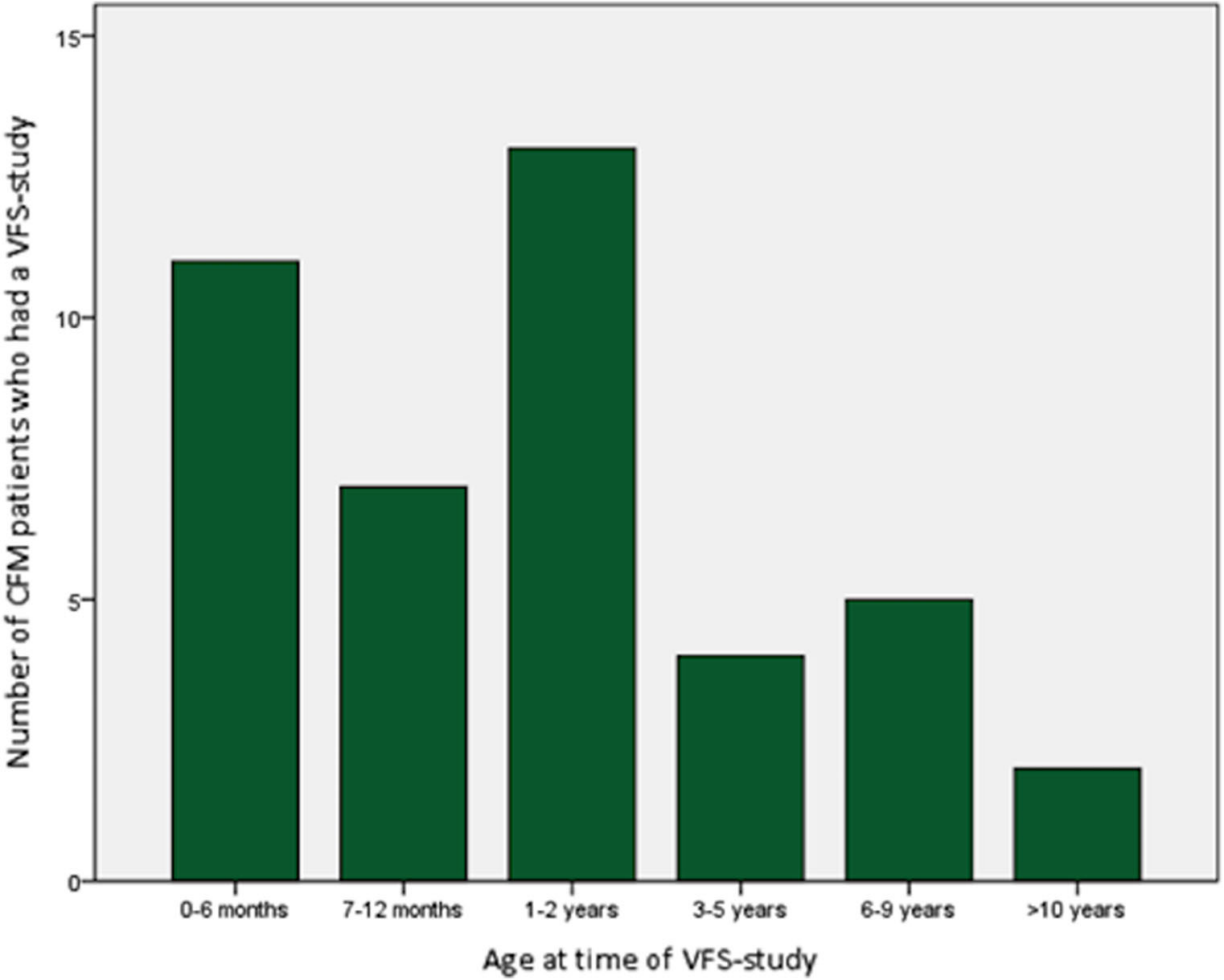
Age at time of first videofluoroscopic swallow study in patients with CFM. *VFS-study* videofluoroscopic swallow study

The majority of patients younger than 6 months showed problems in all phases of the VFS-study; most problems were seen in the bolus formation (62.5%), nasopharyngeal reflux (75%), and aspiration (62.5%). Patients younger than 6 months were significantly more often diagnosed with nasopharyngeal reflux than patients older than 6 months (Pearson’s *χ* ^2^ (1) = 7.529, *p* = 0.011). The group of patients older than 6 months (*n* = 31) showed mostly inappropriate bolus formation (55%), delayed/variable swallow trigger (47.4%), and post-swallow stasis (47.1%) (Fig. 2 and Table 4).

**Table 4:** Outcome of the VFS-study before or after the age of 6 months

	Age at time of VFS-study		
	< 6 months	> 6 months	Total
Oral phase			
Inappropriate bolus formation	5 out of 8 (62.5%)	11 out of 20 (55.0%)	16 out of 28 (57.1%)
Premature spill into the pharynx	4 out of 8 (50.0%)	3 out of 16 (18.7%)	7 out of 24 (29.2%)
Pharyngeal phase			
Delayed/variable swallow trigger	4 out of 7 (57.0%)	9 out 19 (47.4%)	13 out of 26 (50.0%)
Post-swallow stasis	3 out of 7 (42.9%)	8 out of 17 (47.1%)	11 out of 24 (45.8%)
Nasopharyngeal reflux	6 out of 8 (75.0%)	4 out of 20 (20.0%)	10 out 28 (35.7%)
Laryngeal penetration	4 out of 7 (57.0%)	4 out of 19 (21.2%)	8 out of 26 (30.8%)
Aspiration	5 out of 8 (62.5%)	5 out of 21 (23.8%)	10 out of 29 (34.5%)

* Numbers do not add up due to unknown outcome of VFS-study

**Table 5:** Overview oral and pharyngeal phase in CFM patients with repaired cleft (lip) palate

CFM patients with repaired cleft (lip) palate	*N*
Oral phase (*n* = 6)	
Inappropriate bolus formation	4
Premature spill into the pharynx	4
Pharyngeal phase (*n* = 7)	
Delayed/variable swallow trigger	6
Post-swallow stasis	4
Nasopharyngeal reflux	4
Laryngeal penetration	3
Aspiration	1

#### The Videofluoroscopic Swallow Study in CFM Patients Without Cleft

##### The Oral Phase (Supplemental Table 1)

Appropriate bolus formation was mostly seen with the use of puree (78.9%, *n* = 15). Inappropriate bolus formation was mostly seen with the use of thin (48.0%, *n* = 12) or thick (47.1%, *n* = 8) liquids. Premature spill into the pharynx was seen when both thin liquids (27.3%, *n* = 6) and thick liquids (23.5%, *n* = 4) were given.

##### The Pharyngeal Phase (Supplemental Tables 2, 3, 4, 5)

The pharyngeal phase included swallow trigger, post-swallow stasis, nasopharyngeal reflux, laryngeal penetration, and aspiration. Overall, and regardless of the consistency used, swallow trigger was tested in 26 patients of which in total 13 patients (50.0%) showed an abnormal swallow trigger. However, when the consistency used was taken into account, delayed swallow trigger was seen in 10.0–33.3% of the patients; the thinner the consistency, the more delayed the swallow trigger. Overall, post-swallow stasis was diagnosed in 45.8% of the tested patients (*n* = 24), but was mostly seen when thick liquids (35.7%, *n* = 5) and puree (35.3%, *n* = 6) were given.

The highest incidence of nasopharyngeal reflux and laryngeal penetration was seen with the use of thin liquids (40.0%, *n* = 10) and thick liquids (35.3%, *n* = 6), and was not seen with the use of solid pellets.

Overall, aspiration was diagnosed in 34.5% of the patients (*n* = 29), regardless of the consistency used. Aspiration was especially seen when thin liquids were used (38.5%, *n* = 10), and three of these patients showed silent aspiration.

##### The Pruzansky–Kaban Classification and the Risk for Swallow Difficulties (Tables 6 and 7)

Inappropriate bolus formation was significantly more often diagnosed in patients with Pruzansky–Kaban III classification than in patients with a lower Pruzansky–Kaban classification (Pearson’s *χ* ^2^(3) = 10.708, *p* = 0.013). However, severe and less severely affected patients were comparably affected in the pharyngeal phase. Furthermore, the outcome of the VFS-studies performed in patients with bilateral CFM (*n* = 9) was not significantly different from patients with unilateral CFM (*n* = 20).

**Table 6:** Pruzansky–Kaban classification of included patients and outcome of the tested phases of the VFS-studies

	P–K I	P–K IIA	P–K IIB	P–K III	P–K unknown	Total
	*n* = 5	*n* = 5	*n* = 4	*n* = 10	*n* = 5*	
Oral phase						
Inappropriate bolus formation	2	2	0	9	3	16
Premature spill into the pharynx	0	3	0	2	2	7
Pharyngeal phase						
Delayed/variable swallow trigger	2	4	0	4	3	13
Post-swallow stasis	1	4	0	4	2	11
Nasopharyngeal reflux	0	2	0	5	3	10
Laryngeal penetration	1	3	0	1	3	8
Aspiration	0	2	2	3	3	10

*P–K* Pruzansky–Kaban classification

* Not included in statistical analyses

**Table 7:** Laterality of craniofacial microsomia and outcome of the tested phases of the VFS-studies

	Unilateral CFM	Bilateral CFM	Total
Oral phase			
Inappropriate bolus formation	1		16
Premature spill into the pharynx	5	2	7
Pharyngeal phase			
Delayed/variable swallow trigger	10	3	13
Post-swallow stasis	6	5	11
Nasopharyngeal reflux	7	3	10
Laryngeal penetration	5	3	8
Aspiration	6	4	10

*CFM* craniofacial microsomia

##### Current Nutritional Route and the Risk for Swallow Difficulties (Supplemental Table 6)

Twenty-one patients were fully orally fed at the time of the VFS-study, five orally in combination with a nasogastric tube and three solely via a nasogastric tube. Current nutritional route did not significantly correlate with the outcome of the VFS-studies in this study.

#### The Videofluoroscopic Swallow Study in CFM Patients with Cleft

Table 5 shows the VFS-study findings of CFM patients with repaired cleft (lip) palate at time of the VFS-study (*n* = 7). The oral phase and pharyngeal phase were affected in these patients.

The oral phase was affected in 4 patients, 4 patients showed ‘inappropriate bolus formation,’ and 4 patients showed ‘premature spill into the pharynx.’ Six out of 7 patients had problems with timing of swallowing, 4 patients showed post-swallow stasis, and 4 showed nasopharyngeal reflux. Laryngeal penetration was seen in 3 patients, but aspiration only in one patient.

## Discussion

By combining the data of three major craniofacial centers, the medical charts of 755 patients were analyzed. In our cohort, 13.5% of the patients were diagnosed with a swallowing disorder, necessitating a VFS-study in 50.9% of these patients. In total, 42 VFS-studies were included for analysis.

The majority of CFM patients with SD, who did not need further examination in the form of a VFS-study, are most likely affected with clinically less relevant SD since there were no clinical concerns for aspiration according to the medical charts. The SD of these patients might resolve by developing compensatory mechanisms and/or by offering smaller volumes with the use of simple adjustments, e.g., Habermann nipple and Dr. Brown’s bottle [22]. The indication for a VFS-study was made by their physician based on clinical symptoms; however, the exact criteria used in the three institutions remain unclear.

In healthy infants, reflexive activities play a key role in swallowing during the first 6 months of life as the brain is still developing [22]. In this study, a considerable number of patients (26.2%) had undergone a VFS-study before the age of 6 months and showed most difficulties in the pharyngeal phase, i.e., nasopharyngeal reflux, laryngeal penetration, and aspiration. Nasopharyngeal reflux, which is considered to be a pathological entity after the age of 3 months, was diagnosed in a considerable number of patients, i.e., in both patients younger and older than 6 months [29, 30]. As our results are based on patients without cleft (lip) palate, it is suggested that the presence of nasopharyngeal reflux in our cohort could be the result of velopharyngeal insufficiency or a neurological disorder [30, 31].

The majority of the patients were evaluated after the age of 6 months (75.6%). Difficulties of bolus formation, timing of swallow trigger, and post-swallow stasis were seen in a relatively smaller number of patients after the age of 6 months. Inappropriate bolus formation, mostly seen in patients with type III mandibular deformities, is likely the result of anatomical anomalies leading to ineffective lip closure, tongue movements/incoordination, or muscle weakness, which was also concluded by Huisinga-Fischer [21]. Yet, it is impossible to rule out differences in innervation and muscle function as (part of) the cause for these problems [7, 22, 23].

In the newborn infant, the pharynx follows a gentle curve from the nasopharynx to the hypopharynx. Growth results in increased anteroposterior dimension of the nasopharynx and an increased angle between the nasopharynx and oropharynx, gradually up to 90° [16, 22, 32]. Difficulties of the pharyngeal phase were seen in a greater number of patients before the age of 6 months than after the age of 6 months. Nasopharyngeal reflux and difficulties with laryngeal penetration and aspiration occurred more often before the age of 6 months. Delayed swallow trigger and post-swallow stasis occurred equally in patients younger and older than 6 months. Moreover, premature spill into the pharynx was seen after the age of 6 months in a smaller number of patients. Even though the nature of triggering the pharyngeal phase of swallowing is relatively unknown, and although the oral and pharyngeal cavities are anatomically apart, it is known that their function is integrated [14, 33, 34]. In these infants, a significant part of the problems might resolve over time. To support this theory, follow-up of VFS-studies is essential to compare the findings over time within this patient group.

A substantial number of patients (31.0%) of the studied cohort also had a cleft (lip) palate. FD and SD seen in these patients might be more complicated in the presence of other craniofacial anomalies [22, 35]. Therefore, patients with CFM and repaired cleft (lip) palate were analyzed separately in this study. Like patients without cleft (lip) palate, not only difficulties were seen in bolus formation and timing of the swallow trigger, but also in the pharyngeal phase. Kaufman et al. found that abnormalities seen in the pharyngeal phase cannot be explained by the presence of cleft (lip) palate and might be the result of hypoplasia of the pharyngeal muscles, which is part of the anomalies seen in CFM [7, 11, 35]. From this study, it cannot be concluded that patients with CFM and cleft (lip) palate have more severe SD than those without cleft (lip) palate. However, patients with CFM and cleft (lip) palate are more frequently NG tube dependent, which influences the development of normal swallowing. However, it should be taken into account that these NG tube-depending patients might be more prone to have SD as a result of the additional anatomical deformities caused by cleft. With regard to the SD, these patients should be seen as a different entity.

Aspiration was tested in all patients and overall diagnosed in 34.5% of the patients (including 4 patients with silent aspiration), regardless of the consistency used, but specifically with thin liquids. This could partly be explained by inappropriate bolus formation which is more frequently seen in patients with CFM and difficulties with timing of swallowing. Whereas patients before the age of 6 months showed aspiration in 62.5% of the cases, aspiration was seen in 23.8% of the cases after the age of 6 months. It is expected that aspiration might resolve when patients have developed compensating mechanisms forming appropriate boluses later in life. Moreover, some studies that analyzed SD in patients with Robin Sequence—a disorder characterized by micrognathia, glossoptosis, and upper airway obstruction—showed that the difficulties seen were proportional to the degree of airway obstruction seen in these patients [36]. Upper airway obstruction is also seen in patients with CFM and therefore it cannot be excluded that a component of airway problems in these infants might (also) play a role in the etiology of SD in CFM [6].

### Limitations

Accuracy of VFS-study interpretation is critical and findings from VFS-studies can be discussed from a variety of viewpoints. Since there is limited research on the interpretation of VFS-study findings in the pediatric population—no criterion-referenced outcome of VFS-study exist for this age group—the results of this study are based on the radiologist’s experience and expertise. A more objective and validated scale for adults does exist for interpreting VFS-study findings: a modified barium swallowing tool used for quantification of swallowing impairment (MBSImp) [37]. With concerns to penetration and aspiration, a Penetration–Aspiration Scale according to Rosenbek (an 8-point scale) exists for adults [38, 39]. The criteria used in these scales are congruent to the VFS-study findings used in this study; however, not all criteria used were identical. Therefore, this study could not benefit from these scales.

To perform the VFS-study, different consistencies were used as a bolus, but no data on the volume of the bolus were available. Literature shows that as bolus size increases, the pharyngeal transit time, laryngeal closure, and elevation increase [40, 41]. However, the included VFS-studies were performed in large craniofacial centers with experienced physicians and the VFS-studies were performed in a standardized setting. Bolus formation can best be imaged with ultrasound and the VFS-studies are ideally performed in a standardized setting and examined by an experienced radiologist [22]. To gain more insight into the pathogenesis of SD in CFM, all patients with SD should undergo a VFS-study because it permits visualization of bolus flow in relation to structural movement throughout the upper aerodigestive tract in real time. In this study, the severity of SD was not included as it was not the aim of the study. The main question is whether a child can swallow safely and successfully.

For clinicians, treatment of FD and SD should preferably be started early in life. Therefore, it is recommended to have all patients with CFM screened for SD by a speech and language therapist and to perform a VFS-study in patients with a type III Pruzansky–Kaban classification or with a high risk for SD after screening by a speech and language therapist. This study shows a trend between the severity of CFM and the outcome of VFS-studies: more severely affected patients show more difficulties with bolus formation and in the pharyngeal phase than less severely affected patients. Possibly, a combination of neuromuscular deficits and anatomical anomalies causes SD seen in patients with CFM.

## Electronic supplementary material

Below is the link to the electronic supplementary material.

Supplementary material 1 (DOCX 16 kb)

Supplementary material 2 (DOCX 16 kb)

Supplementary material 3 (DOCX 15 kb)

Supplementary material 4 (DOCX 16 kb)

Supplementary material 5 (DOCX 16 kb)

Supplementary material 6 (DOCX 15 kb)
