# Supplementary material for: Evaluation of Swallow Function in Patients with Craniofacial Microsomia: A Retrospective Study
Source: Dysphagia. 2017 Nov 4;33(2):234–42. doi: 10.1007/s00455-017-9851-x (PMC5866261; doi:10.1007/s00455-017-9851-x)
Supplement: Supplementary file 1 — Supplementary material 1 (DOCX 16 kb) [file 455_2017_9851_MOESM1_ESM.docx]

| **Oral phase**  **Consistencies** | **Thin** | | **Thick** | | **Puree** | | | **Solids** | |
| --- | --- | --- | --- | --- | --- | --- | --- | --- | --- |
| **Bolus** **Formation** | n | % | n | % | n | % | | n | % |
| **Appropriate** | 12 | 48,0 | 9 | 52,9 | 15 | 78,9 | | 6 | 60,0 |
|  |  |  |  |  |  |  | |  |  |
| **Inappropriate** | 12 | 48,0 | 8 | 47,1 | 3 | 15,8 | | 4 | 40,0 |
|  |  |  |  |  |  |  | |  |  |
| **Noncompliance** | 1 | 4,0 | -- | -- | 1 | 5,3 | | -- | -- |
| **Total** | 25 | 100,0 | 17 | 100,0 | 19 | 100,0 | 10 | | 100,0 |
|  |  |  |  |  |  |  | |  |  |
| **Premature spill into pharynx** | n | % | n | % |  |  | |  |  |
| **No** | 16 | 72,7 | 13 | 76,5 | N/A | N/A | | N/A | N/A |
|  |  |  |  |  |  |  | |  |  |
| **Yes** | 6 | 27,3 | 4 | 23,5 | N/A | N/A | | N/A | N/A |
|  |  |  |  |  |  |  | |  |  |
| **Total** | 22 | 100,0 | 17 | 100,0 |  |  | |  |  |

Supplemental table 1. Oral phase of VFS-study.

N/A = not applicable
