# Supplementary material for: Evaluation of Swallow Function in Patients with Craniofacial Microsomia: A Retrospective Study
Source: Dysphagia. 2017 Nov 4;33(2):234–42. doi: 10.1007/s00455-017-9851-x (PMC5866261; doi:10.1007/s00455-017-9851-x)
Supplement: Supplementary file 2 — Supplementary material 2 (DOCX 16 kb) [file 455_2017_9851_MOESM2_ESM.docx]

| **Pharyngeal phase**  **Consistencies** | **Thin** | | **Thick** | | **Puree** | | **Solids** | |
| --- | --- | --- | --- | --- | --- | --- | --- | --- |
| **Swallow trigger** | n | % | n | % | n | % | n | % |
| **Timely** | 13 | 54,2 | 11 | 68,8 | 16 | 84,2 | 9 | 90,0 |
|  |  |  |  |  |  |  |  |  |
| **Variable** | 2 | 8,3 | 1 | 6,3 | -- | -- | -- | -- |
|  |  |  |  |  |  |  |  |  |
| **Delayed** | 9 | 33,3 | 4 | 25,0 | 2 | 10,5 | 1 | 10,0 |
|  |  |  |  |  |  |  |  |  |
| **No** **initiation** | -- | -- | -- | -- | 1 | 5,3 | 0 | 0 |
|  |  |  |  |  |  |  |  |  |
| **Total** | 24 | 100,0 | 16 | 100,0* | 19 | 100,0 | 10 | 100,0 |

Supplemental table 2. Results swallow trigger (pharyngeal phase) of VFS-studies.
*Numbers do not add up due to rounding numbers.
