# Supplementary material for: Evaluation of Swallow Function in Patients with Craniofacial Microsomia: A Retrospective Study
Source: Dysphagia. 2017 Nov 4;33(2):234–42. doi: 10.1007/s00455-017-9851-x (PMC5866261; doi:10.1007/s00455-017-9851-x)
Supplement: Supplementary file 3 — Supplementary material 3 (DOCX 15 kb) [file 455_2017_9851_MOESM3_ESM.docx]

| **Pharyngeal phase**  **Consistencies** | **Thin** | | **Thick** | | **Puree** | | **Solids** | |
| --- | --- | --- | --- | --- | --- | --- | --- | --- |
| **Post-swallow stasis** | n | % | n | % | n | % | n | % |
| **Yes** | 6 | 27,3 | 5 | 35,7 | 6 | 35,3 | 3 | 30,0 |
|  |  |  |  |  |  |  |  |  |
| **No** | 16 | 72,7 | 9 | 64,3 | 11 | 64,7 | 7 | 70,0 |
|  |  |  |  |  |  |  |  |  |
| **Total** | 22 | 100,0 | 14 | 100,0 | 17 | 100,0 | 10 | 100,0 |

Supplemental table 3. Results post swallow stasis (pharyngeal phase) of VFS-studies.
