# Supplementary material for: Evaluation of Swallow Function in Patients with Craniofacial Microsomia: A Retrospective Study
Source: Dysphagia. 2017 Nov 4;33(2):234–42. doi: 10.1007/s00455-017-9851-x (PMC5866261; doi:10.1007/s00455-017-9851-x)
Supplement: Supplementary file 4 — Supplementary material 4 (DOCX 16 kb) [file 455_2017_9851_MOESM4_ESM.docx]

| **Pharyngeal phase**  **Consistencies** | **Thin** | | **Thick** | | **Puree** | | **Solids** | |
| --- | --- | --- | --- | --- | --- | --- | --- | --- |
| **Nasopharyngeal reflux** | n | % | n | % | n | % | n | % |
| **Yes** | 10 | 40,0 | 6 | 35,3 | 3 | 15,8 | N/A | N/A |
|  |  |  |  |  |  |  |  |  |
| **No** | 15 | 60,0 | 11 | 64,7 | 16 | 84,2 | 10 | 100 |
|  |  |  |  |  |  |  |  |  |
| **Total** | 25 | 100,0 | 17 | 100,0 | 19 | 100,0 | 10 | 100 |
|  |  |  |  |  |  |  |  |  |
| **Laryngeal penetration** | n | % | n | % | n | % | n | % |
| **Single** **episode** | 2 | 8,3 | -- | -- | -- | -- | N/A | N/A |
|  |  |  |  |  |  |  |  |  |
| **Multiple** | 5 | 20,8 | 4 | 23,5 | 1 | 5,3 | N/A | N/A |
|  |  |  |  |  |  |  |  |  |
| **No** | 17 | 70,8 | 13 | 76,5 | 18 | 94,7 | 10 | 100 |
|  |  |  |  |  |  |  |  |  |
| **Total** | 24 | 100,0 | 17 | 100,0 | 19 | 100,0 | 10 | 100 |

Supplemental table 4. Results post swallow stasis (pharyngeal phase) of VFS-studies.
N/A = not applicable.
