# Supplementary material for: Evaluation of Swallow Function in Patients with Craniofacial Microsomia: A Retrospective Study
Source: Dysphagia. 2017 Nov 4;33(2):234–42. doi: 10.1007/s00455-017-9851-x (PMC5866261; doi:10.1007/s00455-017-9851-x)
Supplement: Supplementary file 5 — Supplementary material 5 (DOCX 16 kb) [file 455_2017_9851_MOESM5_ESM.docx]

| **Pharyngeal phase**  **Consistencies** | **Thin** | | **Thick** | | **Puree** | | **Solids** | |
| --- | --- | --- | --- | --- | --- | --- | --- | --- |
| **Aspiration** | n | % | n | % | n | % | n | % |
| **Yes** | 10 | 38,5 | 5 | 27,8 | 1 | 5,0% | N/A | N/A |
| **Micro-aspiration** | 2 | 7,7 | 1 | 5,6 | -- | -- | N/A | N/A |
| **Silent aspiration** | 3 | 11,5 | 2 | 11,1 | -- | -- | N/A | N/A |
| **Not further specified** | 5 | 19,2 | 2 | 11,1 | 1 | 5,0 | N/A | N/A |
|  |  |  |  |  |  |  |  |  |
| **No** | 16 | 61,5 | 13 | 72,2 | 19 | 95,0 | 10 | 100 |
|  |  |  |  |  |  |  |  |  |
| **Total** | 26 | 100,0 | 18 | 100,0 | 20 | 100,0 | 10 | 100,0 |
|  |  |  |  |  |  |  |  |  |

Supplemental table 5. Results of aspiration and airway protection (pharyngeal phase) of VFS-studies.
N/A = not applicable.
