# Supplementary material for: Evaluation of Swallow Function in Patients with Craniofacial Microsomia: A Retrospective Study
Source: Dysphagia. 2017 Nov 4;33(2):234–42. doi: 10.1007/s00455-017-9851-x (PMC5866261; doi:10.1007/s00455-017-9851-x)
Supplement: Supplementary file 6 — Supplementary material 6 (DOCX 15 kb) [file 455_2017_9851_MOESM6_ESM.docx]

|  | **Oral**  **n = 21** | **Oral & tube  n=5** | **Tube**  **n=3** | **Total** |
| --- | --- | --- | --- | --- |
| **Oral phase**   - Inappropriate bolus formation - Premature spill into the pharynx | 11  6 | 5  1 | 0  0 | 16  7 |
| **Pharyngeal phase**   - Delayed/variable swallow trigger - Post-swallow stasis - Nasopharyngeal reflux - Laryngeal penetration - Aspiration | 8  6  8  6  8 | 3  3  2  2  2 | 2  2  0  0  0 | 13  11  10  8  10 |

Supplemental table 6. Nutritional route and outcome of the tested phases of the VFS-studies.
